# Fatty Acid-Derived Biofuels and Chemicals Production in *Saccharomyces cerevisiae*

**DOI:** 10.3389/fbioe.2014.00032

**Published:** 2014-09-01

**Authors:** Yongjin J. Zhou, Nicolaas A. Buijs, Verena Siewers, Jens Nielsen

**Affiliations:** ^1^Department of Chemical and Biological Engineering, Chalmers University of Technology, Gothenburg, Sweden

**Keywords:** fatty acid metabolism, fatty acid ethyl ester, fatty alcohol, alkanes/alkene, yeast

## Abstract

Volatile energy costs and environmental concerns have spurred interest in the development of alternative, renewable, sustainable, and cost-effective energy resources. Environment-friendly processes involving microbes can be used to synthesize advanced biofuels. These fuels have the potential to replace fossil fuels in supporting high-power demanding machinery such as aircrafts and trucks. From an engineering perspective, the pathway for fatty acid biosynthesis is an attractive route for the production of advanced fuels such as fatty acid ethyl esters, fatty alcohols, and alkanes. The robustness and excellent accessibility to molecular genetics make the yeast *Saccharomyces cerevisiae* a suitable host for the purpose of bio-manufacturing. Recent advances in metabolic engineering, as well as systems and synthetic biology, have now provided the opportunity to engineer yeast metabolism for the production of fatty acid-derived fuels and chemicals.

## Introduction

Volatile energy costs and environmental concerns have motivated the development of sustainable, renewable, and cost-effective alternative energy sources that have reduced pollution emissions or carbon footprints (Fortman et al., 2008). Biofuels are such green alternatives to petroleum-based fuels, given the capacity of photosynthetic organisms to recycle CO_2_ after biofuel combustion, thereby leading to near-zero net greenhouse gas emissions. Microbial synthesis is an attractive approach for biofuel production due to the large flexibility for pathway engineering and low environmental footprint. Among the metabolic pathways, fatty acid biosynthesis has attracted significant attention for production of highly reduced biofuels and chemicals with high energy densities (Lennen and Pfleger, 2013).

The yeast *Saccharomyces cerevisiae* is a well-studied model microorganism and is particularly suited for commercial scale processes due to its robustness and tolerance toward industrial conditions, the capability of high-density fermentations, and insusceptibility toward phage contamination (Nielsen et al., 2013; Mattanovich et al., 2014). In fact, *S. cerevisiae* has been successfully applied for the industrial production of bioethanol (Mussatto et al., 2010). Additional studies have shown that it can be engineered for the production of a variety of fuel molecules including isobutanol, butanol, and farnesene (Buijs et al., 2013). Recently, it has received increased attention as a host for the synthesis of fatty acid-derived biofuels and chemicals (Li et al., 2014; Runguphan and Keasling, 2014). This review will summarize recent progress in the engineering of *S. cerevisiae* for the production of fatty acid-derived biofuels and chemicals, in addition to analyzing the current challenges in increasing their productivity for commercial deployment.

## Fatty Acid Production in *S. cerevisiae*

Free fatty acids (FFAs) can be used for the industrial manufacturing of detergents, soaps, lubricants, cosmetics, and pharmaceutical ingredients (Tee et al., 2014). In addition, FFAs can also serve as precursors for the production of alkanes by catalytic decarboxylation (Lennen et al., 2010) or fatty acid methyl esters (FAMEs) through esterification (Christie and Han, 2010).

**Figure 1:**
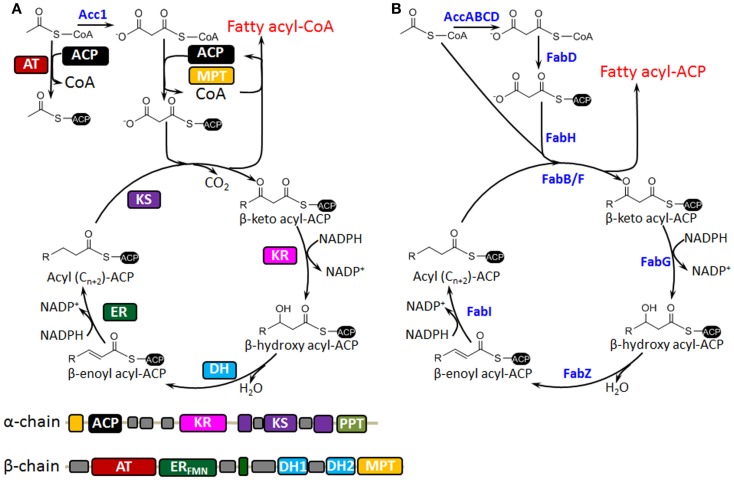
**Comparison of *S. cerevisiae* type I (A) and bacterial type II (B) fatty acid synthases is shown**. **(A)** The catalytic reaction cycle of and domain organization of yeast fatty acid synthase. Acetyl-CoA is activated by ACP acyltransferase (AT) and then malonyl-CoA is iteratively fed into the reaction cycle by malonyl/palmitoyl transferase (MPT). The elongation process is consecutively catalyzed by ketoacyl reductase (KR), dehydratase (DH), and enoyl reductase (ER). After several rounds of elongation, the end product is released from the enzyme as a fatty acyl-CoA after back-transfer to CoA from ACP by the double-functional MPT. Desaturation of fatty acyl-CoA takes place in the endoplasmic reticulum and is catalyzed by the Δ9-fatty acid desaturase Ole1, and very long-chain fatty acids (VLFA) are synthesized by chain elongation of saturated acyl-CoAs through a cyclic series of reactions reminiscent of fatty acid *de novo* synthesis. **(B)** Bacterial type II fatty acid synthase (FAS) that consists of discrete, monofunctional enzymes. Acetoacetyl-ACP is in prior synthesized for the initiation of chain elongation, and then malonyl-CoA is iteratively fed into the elongation cycle after ACP loading, which is catalyzed by FabD. Desaturation can be performed at the C10 chain length by 3-hydroxydecanoyl-ACP dehydrase (FabA), and the product *cis*-3-enoyl acyl-ACP bypasses FabI of reduction and goes to FabB for the next round of elongation. Different from yeast fatty acid biosynthesis, the end product is released as a fatty acyl-ACP after several rounds of elongation.

The biosynthesis of fatty acids in *S. cerevisiae* differs from that in bacteria such as *Escherichia coli* (Figure 1). In bacteria, fatty acid synthesis is carried out by a type II fatty acid synthase (FAS) that consists of discrete, monofunctional enzymes (Figure 1B); while in *S. cerevisiae*, the *de novo* synthesis of fatty acids can take place in at least two subcellular compartments: cytoplasm (type I FAS) and mitochondria (type II FAS). Mitochondrial FAS II has been implicated as the sole mitochondrial source of octanoic acid, which is a precursor of the lipoic acid (LA) cofactor that is required for maintaining the function of several mitochondrial enzyme complexes such as pyruvate dehydrogenase (Hiltunen et al., 2009). However, most functional and storage lipids are synthesized by cytosol type I FAS (Koch et al., 2014), which is a large, multifunctional dimeric complex that is responsible for fatty acid synthesis from malonyl-CoA and acetyl-CoA (Figure 1A). This distinction is important as it has implications for further metabolic engineering of fatty acid metabolism. Considering its predominant role for fatty acids synthesis, we will focus on the FAS I system. This process starts with loading of acetyl-CoA to the acyl carrier protein (ACP) by the ACP acyltransferase (AT). Then consecutive catalytic steps of β-ketoacyl-ACP synthesis, β-ketoacyl-ACP reduction, β-hydroxyacyl-ACP dehydration, and enoyl-ACP reduction extend the chain length in a repetitive manner by using malonyl-CoA as building blocks. The malonyl-CoA is synthesized from acetyl-CoA by incorporation of CO_2_, which is catalyzed by acetyl-CoA carboxylase (Acc1). The chain extension usually stops at palmitoyl-ACP after seven cycles, which is mainly determined by the ketoacyl synthase domain (Sangwallek et al., 2013). Finally, acyl-ACP and malonyl-CoA are transformed by malonyl transacylase (MPT) to form acyl-CoA and the activated malonyl-ACP, which is necessary for initiating the next acyl-CoA synthesis. Acyl-CoA can be transformed into lipids or FFAs catalyzed by AT or thioesterase, respectively.

Disruption of two main fatty acyl-CoA synthetase-encoding genes *FAA1* and *FAA4* led to the production of 900 μmol/L (≈240 mg/L) FFAs, of which 220 μmol/L (≈60 mg/L) FFAs were secreted. The accumulation of FFAs was most likely the result of interrupted lipid remodeling processes (Scharnewski et al., 2008). Besides *FAA1* and *FAA4* disruption, overexpression of the FAS subunits (Fas1 and Fas2), acetyl-CoA carboxylase (Acc1), and *E. coli* acyl-ACP thioesterase (TesA) resulted in a higher FFAs production of 400 mg/L. Blockage of β-oxidation was also beneficial for FFA accumulation though it was found to be much less effective than elimination of fatty acyl-CoA synthetase (Runguphan and Keasling, 2014). Similarly, after disruption of the β-oxidation pathway, (i) elimination of the acyl-CoA synthetases, (ii) overexpression of different thioesterases, and (iii) enhancement of acetyl-CoA/malonyl-CoA, the engineered strain produced more than 140 mg/L FFAs (Li et al., 2014). Additionally, acetyl-CoA carboxylase (Acc1) was identified as a critical bottleneck for fatty acid synthesis in *S. cerevisiae* with a cell-free system (Li et al., 2014).

**Figure 2:**
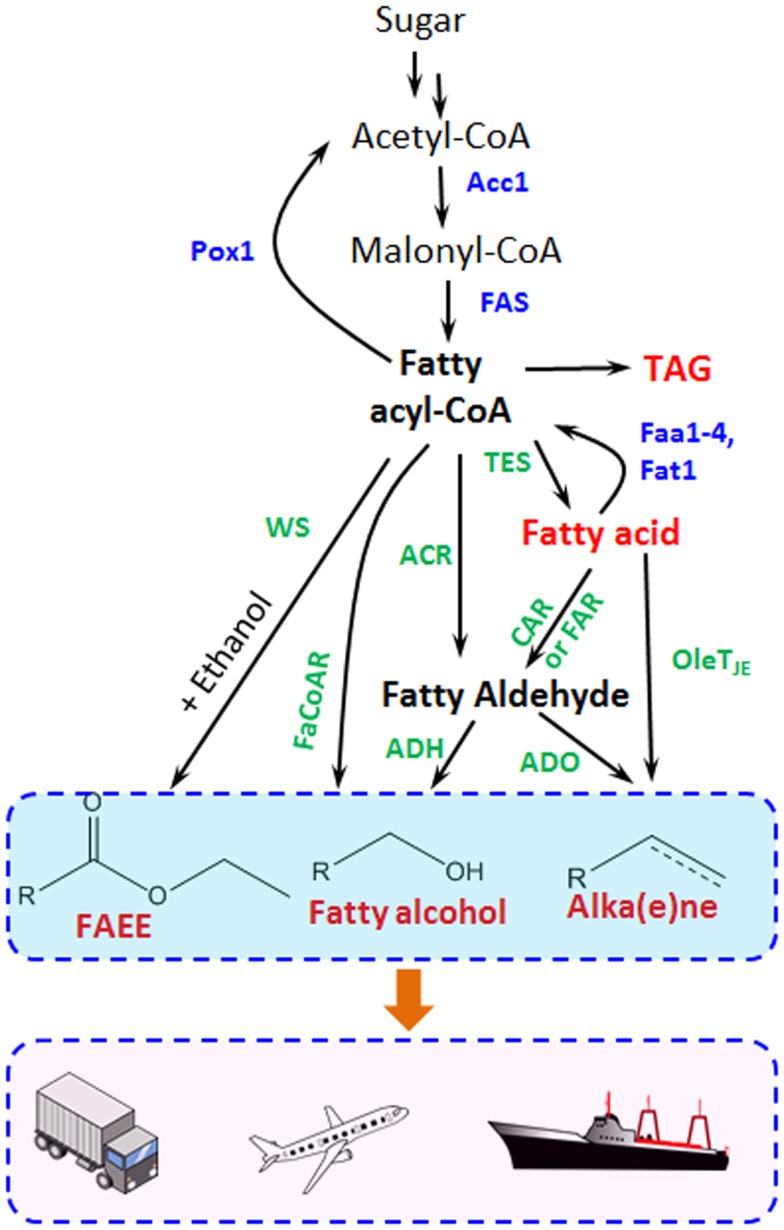
**Metabolic pathways for fatty acid-derived biofuel and chemical biosynthesis are shown**. Acc1, acetyl-CoA carboxylase; FAS, fatty acid synthase; Pox1, acyl-CoA oxidase; Faa1-4 and Fat1, acyl-CoA synthetase; TES, thioesterase; ACR, fatty acyl-CoA reductase for fatty aldehyde synthesis; FaCoAR, fatty acyl-CoA reductase for fatty alcohol production; CAR, carboxylic acid reductase; FAR, fatty acid reductase; WS, wax ester synthase; ADO, aldehyde-deformylating oxygenase; ADH, alcohol dehydrogenase. The blue marked enzymes responsible for the endogenous fatty acid metabolism, while the green ones transform fatty acids and intermediates to different biofuels and oleo-chemicals.

Fatty acid metabolism has also been widely tailored for producing fatty acid ethyl esters (FAEEs), fatty alcohols, and alkanes/alkenes with different chain length in prokaryotes, especially in *E. coli*, which has been reviewed elsewhere (Lennen and Pfleger, 2013; Janssen and Steinbuchel, 2014). Figure 2 illustrates how the different fatty acid-derived products can be obtained in yeast and is described in greater detail within the subsequent sections.

## Triacylglycerol Production

Besides FFAs, microbial lipids have also been attracting great attention as alternative feedstocks to vegetable oils and animal fats for production of FAMEs that are used as biodiesel (Hu et al., 2011). Triacylglycerols (TAGs), which can accumulate to very high levels in eukaryotic cells, play an essential role in energy storage (Sorger and Daum, 2003; Klug and Daum, 2014). Wild-type *S. cerevisiae* strains typically do not accumulate storage lipids to more than 10% dry cell weight (DCW), whereas some oleaginous yeasts and fungi can accumulate TAGs to much higher levels (Li et al., 2007). Overexpression of several genes encoding FAS (*FAS1* and *FAS2*), acetyl-CoA carboxylase (*ACC1*), and diacylglycerol AT (*DGA1*) made *S. cerevisiae* BY4742 accumulate total TAGs to 17% DCW (Runguphan and Keasling, 2014). Mimicking the metabolism of oleaginous microorganisms, by introduction of ATP-citrate lyase (ACL) and disruption of isocitrate dehydrogenase genes *IDH1* and *IDH2*, increased the total fatty acid content by 21% (Tang et al., 2013). From transposon mutagenesis analysis, it was found that disruption of the transcription factor Snf2 resulted in a 70% increase of TAGs (Kamisaka et al., 2006). Furthermore, when the fatty acyl-CoA synthetase-encoding gene *FAA3* and the diacylglycerol AT-encoding gene *DGA1* were overexpressed, total lipid accumulation increased to 30% DCW, of which TAGs were the most abundant (Kamisaka et al., 2007). Overexpression of the active diacylglycerol AT variant Dga1ΔN, lacking the N-terminal 29 amino acids in the *dga1*Δ and *snf2Δ* mutant, further increased lipid production up to 45% (Kamisaka et al., 2013). This is comparable to levels found in oleaginous yeast and is the highest lipid content reported for *S. cerevisiae* so far. It is worthy to mention that the disruption of *SNF2* (Kamisaka et al., 2007) is more efficient in boosting TAG accumulation than overexpression of fatty acid biosynthesis-related genes (Runguphan and Keasling, 2014), which indicates that TAG and fatty acid biosynthesis are under transcriptional control. The high TAG accumulation provides a feasible alternative route for FAME-based biodiesel production by direct methanolysis of oleaginous microbial biomass (Liu and Zhao, 2007).

## Fatty Acid Ethyl Ester Production

Fatty acid ethyl esters are potentially attractive diesel fuel replacements due to their high energy density and low host toxicity (Zhang et al., 2012). FAEEs can be synthesized by condensation of acyl-CoAs and ethanol by using a wax ester synthase/acyl-CoA:diacylglycerol AT (WS/DGAT). An early attempt at FAEE biosynthesis in *S. cerevisiae* using a WS/DGAT from *Acinetobacter calcoaceticus* ADP1 resulted in a very poor yield (Kalscheuer et al., 2004). Recently, five different wax ester synthases were investigated for their ability to perform FAEE biosynthesis (Shi et al., 2012). It was found that the wax ester synthase from *Marinobacter hydrocarbonoclasticus* had the best performance toward short-chain alcohols *in vitro*. This enzyme also led to production of the highest FAEE titer of 6.3 mg/L when expressed in yeast. In order to overcome the instability and metabolic burden of the plasmid-based enzyme expression, the wax ester synthase gene was integrated, as multiple copies, into the yeast chromosome, and the FAEE production increased sixfold up to 34 mg/L (Shi et al., 2014b).

Through overexpression of *ACC1* encoding acetyl coenzyme A carboxylase (ACCase), FAEE production was further enhanced by 30%, resulting in FAEE production of 8.2 mg/L (Shi et al., 2012). Acetyl-CoA carboxylation is a flux controlling step in fatty acid biosynthesis and overexpression of *ACC1* has been shown to increase fatty acid production by 58% (Runguphan and Keasling, 2014). However, Acc1 activity is strictly regulated by phosphorylation under the control of Snf1. Thus, abolishing phosphorylation regulation of Acc1 by introducing double mutants of S1157A and S659A, increased ACCase activity 3.1-fold in wild-type strain, which was an even higher improvement than *snf1*Δ mutation (Shi et al., 2014a). Overexpression of *ACC1*^S1157A,S659A^ increased FAEEs threefold to 15.8 mg/L, while overexpression of wild-type *ACC1* just led to an increase of 20% (Shi et al., 2014a). This study should be helpful for increasing the production of other malonyl-CoA- and fatty acid-derived chemicals.

In order to enhance the fatty acyl-CoA level for FAEE production, fatty acyl-CoAs consuming pathways, including β-oxidation, sterylesters (SEs), and triacylglycerols (TAG) biosynthesis were blocked. Compared to the wild-type, there was approximately a threefold increase in FAEE production of 17.2 mg/L in the mutant strain. Additionally, the FFAs increased fivefold, which may be helpful for producing other fatty acid-derived molecules (Valle-Rodriguez et al., 2014). Another report showed that enhancing the fatty acid biosynthesis pathway and blocking β-oxidation in *S. cerevisiae* BY4742 increased FAEE production of 4.5-fold (Runguphan and Keasling, 2014). However, the FAEE titer of 5.4 mg/L was less than the strain (17.2 mg/L) in which all the fatty acyl-CoAs consuming pathways were blocked (Valle-Rodriguez et al., 2014), indicating that a high level of fatty acyl-CoAs is essential for efficient FAEE production. Since fatty acyl-CoA biosynthesis requires large amounts of NADPH and acetyl-CoA, the ethanol degradation pathway was therefore up-regulated and a phosphoketolase pathway was introduced to increase acetyl-CoA and NADPH supply for FAEE production (de Jong et al., 2014). By overexpression of the ethanol degradation pathway enzymes alcohol dehydrogenase Adh2, acetaldehyde dehydrogenase Ald6, and the *Salmonella enterica* acetyl-CoA synthetase variant Acs_SE_ (L641P) (Starai et al., 2005), the FAEE yield increased threefold. Introduction of the phosphoketolase pathway yielded a 1.7-fold improvement in FAEE production to 5.1 mg/gDCW in shake flasks (de Jong et al., 2014).

To date, the highest reported FAEE production in *S. cerevisiae* is 0.52 g/L by using glycerol as a carbon source after enhancing ethanol biosynthesis, down-regulating glycerol export, and also adding exogenous fatty acids (Yu et al., 2012). Given that the addition of exogenous fatty acids was essential for high FAEE production, the enhancement of fatty acid biosynthesis would be necessary for the increased *de novo* synthesis of FAEE from sugar-based carbon sources.

## Fatty Alcohol Production

Fatty alcohols are widely used as detergents, skin care products, cosmetics, and medicines and are also considered as potential biofuels (Liu et al., 2014a). Fatty alcohol biosynthesis can proceed via the reduction of a fatty aldehyde intermediate, by an aldehyde reductase. The fatty aldehyde can be generated from (i) fatty acyl-ACP via a fatty acyl-ACP reductase (Schirmer et al., 2010), (ii) acyl-CoA via an acyl-CoA reductase (Reiser and Somerville, 1997), or (iii) fatty acid via a carboxylic acid reductase (Akhtar et al., 2013). Fatty alcohols can also be synthesized directly from fatty acyl-CoA in a four-electron reduction manner catalyzed by a bi-functional fatty acyl-CoA reductase (Willis et al., 2011). Introduction of an NADPH-dependent bi-functional fatty acyl-CoA reductase from *Mus musculus* in wild-type *S. cerevisiae* BY4742 led to fatty alcohol production of 47.4 mg/L. After enhancing the fatty acid biosynthesis pathway and introducing a malic enzyme from *Mucor circinelloides* for improved NADPH supply, the fatty alcohol titer increased to 98 mg/L (Runguphan and Keasling, 2014), which showed the potential of *S cerevisiae* as a platform for fatty alcohol production. Though this titer is not comparable with that reached in *E. coli* of more than 1.5 g/L in fed-batch cultivation (Youngquist et al., 2013), further increase of fatty acid biosynthesis and engineering of more efficient fatty acyl-CoA/fatty acid reduction pathways should be helpful for fatty alcohol overproduction (Akhtar et al., 2013).

## Alkane/Alkene Production

Fatty acid intermediates are ideal precursors for alkane production. Though alkane synthesis in microbes was discovered decades ago, the detailed biochemical pathway for converting fatty acids or their intermediates to alkanes was elucidated only recently (Schirmer et al., 2010). The pathway involves the activities of fatty acyl-ACP reductase and aldehyde-deformylating oxygenase that catalyze the reduction of fatty acyl-ACP to the aldehyde, followed by its conversion to the alkane. Then, two other aldehyde decarbonylases that convert fatty aldehydes into their corresponding *n* − 1 alkanes were identified in *Drosophila melanogaster* (Qiu et al., 2012) and *Arabidopsis thaliana* (Bernard et al., 2012). Several other modification enzymes involved in alkane/alkene biosynthesis have also been identified and characterized including Claisen condensation enzymes (Beller et al., 2010; Sukovich et al., 2010; Frias et al., 2011), a novel cytochrome P450 fatty acid decarboxylase (Rude et al., 2011) and a PKS synthase (Mendez-Perez et al., 2011). Most of these enzymes were heterologously expressed and characterized in *E. coli* for alkane/alkene production (Schirmer et al., 2010; Lennen and Pfleger, 2013; Wang and Lu, 2013; Liu et al., 2014b), and the highest alkane titer reached 580 mg/L by using *Clostridium acetobutylicum* fatty acyl-CoA reductase and *A. thaliana* fatty aldehyde decarbonylase (Choi and Lee, 2013). However, the engineering of alkane production in *S. cerevisiae* is lagging behind. To date, there is only one report concerning alkane production in *S. cerevisiae*, which was achieved via reconstruction of the *A. thaliana* very long-chain alkane biosynthesis pathway (Bernard et al., 2012). Introduction of the aldehyde decarbonylase CER1 and the cognate acyl-CoA reductase CER3 in *S. cerevisiae* INVSur4#, a very long-chain fatty acid producer, enabled synthesis of 19 μg/g DCW very long-chain alkanes, primarily 29 carbons in length. Furthermore, expression of *Arabidopsis* cytochrome *b*_5_ isoforms (CYTB5s) and long-chain acyl-CoA synthetase 1 (LACS1) for enhancing CER1–CER3 activity and precursor supply, respectively, increased alkane production up to 86 μg/g DCW (Bernard et al., 2012). There were no medium-chain alkanes (<20 carbons in length) observed, even though it was recently shown that CER1 is active toward short-chain fatty aldehydes in *E. coli* (Choi and Lee, 2013). The difficulty of alkane production in *S. cerevisiae* may be attributed to the complexity of yeast metabolism, difficulty in expressing complex bacterial enzymes, formation of by-products, and/or alkane toxicity.

In order to overcome the toxicity of alkanes, especially short-chain alkanes, to *S. cerevisiae*, heterologous efflux pumps ABC2 and ABC3 from *Yarrowia lipolytica*, an alkane-assimilating yeast, were shown to significantly increase the tolerance against decane and undecane in *S. cerevisiae* through maintaining lower intracellular alkane levels (Chen et al., 2013). In addition, the endogenous efflux pumps Snq2 and Pdr5 were identified to be involved in alkane export and tolerance by transcriptional analysis of *S. cerevisiae* cells that were exposed to decane and undecane (Ling et al., 2013). These efflux pumps serve as valuable tools for improving the cellular tolerance of yeast toward the production of alkanes.

## Concluding Remarks

There is an urgent need to develop sustainable economic approaches for the production of fuels and other chemicals, traditionally derived from petroleum, using renewable feedstocks. With regard to engineering, the fatty acid biosynthetic pathway is such an attractive target for the production of a wide range of chemicals and transportation fuels. In recent years, several modifying enzymes have been identified for transforming fatty acids and their intermediates to alkanes and alcohols with different chain lengths. So far, the potential of these enzymes for the production of fatty acid-derived biofuels and chemicals has been evaluated extensively in *E. coli* (Lennen and Pfleger, 2013; Janssen and Steinbuchel, 2014). Developments are lagging behind in *S. cerevisiae*, even though this cell factory has been widely used for producing numerous types of chemicals (Hong and Nielsen, 2012; Zhou et al., 2012; Paddon et al., 2013). This is probably due to the more complicated cellular metabolism in addition to its complex regulatory mechanisms and cellular compartmentalization. Nonetheless, given its robustness and the ease with which it can be genetically manipulated, *S. cerevisiae* certainly merits further attention with regard to the production of advanced fuels, as reflected in several recent studies (Li et al., 2014; Runguphan and Keasling, 2014; Shi et al., 2014b).

## Conflict of Interest Statement

The authors declare that the research was conducted in the absence of any commercial or financial relationships that could be construed as a potential conflict of interest.
